# Surrogate endpoints in oncology: when are they acceptable for regulatory and clinical decisions, and are they currently overused?

**DOI:** 10.1186/s12916-017-0902-9

**Published:** 2017-07-21

**Authors:** Robert Kemp, Vinay Prasad

**Affiliations:** 1 School of Medicine, University of Oxford, John Radcliffe Hospital, Oxford, OX3 9DU UK; 2 Division of Hematology Oncology, Knight Cancer Institute, Oregon Health and Science University, 3181 SW Sam Jackson Park Road, Portland, OR 97239 USA; 3 Department of Public Health and Preventive Medicine, Oregon Health and Sciences University, 3181 SW Sam Jackson Park Road, Portland, OR 97239 USA; 4 Center for Health Care Ethics, Oregon Health and Sciences University, 3181 SW Sam Jackson Park Road, Portland, OR 97239 USA

**Keywords:** Surrogate endpoints, Outcomes, Cancer, Regulation, US Food and Drug Administration (FDA)

## Abstract

**Background:**

Surrogate outcomes are not intrinsically beneficial to patients, but are designed to be easier and faster to measure than clinically meaningful outcomes. The use of surrogates as an endpoint in clinical trials and basis for regulatory approval is common, and frequently exceeds the guidance given by regulatory bodies.

**Discussion:**

In this article, we demonstrate that the use of surrogates in oncology is widespread and increasing. At the same time, the strength of association between the surrogates used and clinically meaningful outcomes is often unknown or weak. Attempts to validate surrogates are rarely undertaken. When this is done, validation relies on only a fraction of available data, and often concludes that the surrogate is poor. Post-marketing studies, designed to ensure drugs have meaningful benefits, are often not performed. Alternatively, if a drug fails to improve quality of life or overall survival, market authorization is rarely revoked.

We suggest this reliance on surrogates, and the imprecision surrounding their acceptable use, means that numerous drugs are now approved based on small yet statistically significant increases in surrogates of questionable reliability. In turn, this means the benefits of many approved drugs are uncertain. This is an unacceptable situation for patients and professionals, as prior experience has shown that such uncertainty can be associated with significant harm.

**Conclusion:**

The use of surrogate outcomes should be limited to situations where a surrogate has demonstrated robust ability to predict meaningful benefits, or where cases are dire, rare or with few treatment options. In both cases, surrogates must be used only when continuing studies examining hard endpoints have been fully recruited.

## Background

The ultimate goal of all oncology drugs is to improve patient-centered endpoints. These ‘hard’ endpoints, which are intrinsically valuable to patients, are increased overall survival (OS), improved quality of life (QoL), or both. However, many drugs are approved or used based solely on their ability to improve surrogate endpoints; outcomes that are not inherently meaningful, but aim to predict hard outcomes.

In oncology, the most commonly used surrogates are response rate; a set of criteria characterizing tumor shrinkage; and time to event endpoints, such as progression-free survival (PFS) or recurrence-free survival (RFS). PFS and RFS are composite endpoints where an event is defined as either growth of tumor beyond an arbitrary threshold (progression) or detectable recurrence of disease, or death. While there is debate as to whether PFS is intrinsically meaningful [1], since patients do not feel when they cross the arbitrary threshold of ‘progression,’ we believe that PFS is, strictly speaking, a surrogate.

In this opinion article, we defend the position that surrogate endpoints can and should be used for regulatory or clinical practice decision-making in specific circumstances, but that in current practice, they are used far beyond what is justifiable. The proper use of surrogates should be confined to situations where robust validation studies demonstrate a reliable ability for a change in a specific surrogate to predict changes in meaningful outcomes. Such validation studies are inherently limited to the specific tumor type, setting (adjuvant/metastatic), line of therapy, types of agents (cytotoxic versus targeted drugs), and specific surrogate–outcome pairings. For example, we can consider whether cytotoxic drugs that improve PFS in first-line metastatic colorectal cancer also improve OS. Data supporting such correlations must be judged on the comprehensiveness and completeness of included studies. Surrogates that are not validated may also be used in conditions that are rare, dire, and with few treatment options. In both cases, given the hard lessons of recent oncology history, we request that surrogates are used only after continuing studies measuring hard endpoints have been fully recruited. Our conclusion is based on several factors.

### Surrogate use is widespread

Between 2009 and 2014, the US Food and Drug Administration (FDA) approved drugs for 83 oncology indications: 55 (66%) were approved on the basis of surrogate outcomes, with 31 approved on the basis of response rate, and 24 on the basis of PFS [2]. One hundred percent (25) of accelerated and 51% (30) of traditional approvals were based on treatment effects with surrogate outcomes [2]. Unlike accelerated approval, traditional approvals do not entail further post-marketing commitments for efficacy, and several drugs that have failed to demonstrate any survival gain remain on the market.

### Surrogate use is increasing

The use of surrogates is becoming more common as the primary endpoint of oncology randomized controlled trials (RCTs). Overviews of oncology RCTs between 1995 and 2009 show that between 1995 and 2004, OS was the primary endpoint in 49% of trials, but by 2005 to 2009 it had declined to 36%. Response rate declined by 14% to 6%, but time-to-event endpoints such as PFS more than made up the ground, rising from 26% to 43% of primary outcomes [3].

### When used, the strength of surrogates is often unknown or weak

Despite tremendous advances in genomic and imaging sciences, there is still no perfect surrogate that invariably predicts the endpoint of interest. Thus, all surrogates are judged by their ability to predict changes in hard endpoints.

Several methods have been developed to assess the predictive value of a surrogate. However, the method most suited for regulatory approval is trial-level surrogate validation [4]. Trial-level validation occurs by plotting a change in the surrogate against the change in the hard endpoint across several randomized studies. Each trial serves as one data point. A linear regression analysis is then performed to see if a correlation exists between a change in the surrogate and change in hard endpoint, and to measure the strength of the correlation, quantified by the R^2^_trial_ statistic. Trial-level validation requires a meta-analysis of all trials that have measured the effect of an intervention on both the surrogate and the hard outcome.

Regulatory authorities have typically required verification of a predictive level, both at the individual and trial level. The German Institute for Quality and Efficiency in Health Care (Institut für Qualität und Wirtschaftlichkeit im Gesundheitswesen, IQWIG) provides guidelines for interpreting the R^2^_trial_ value (Table 1), indicating cut-offs where surrogates are deemed suitable for regulatory use. These values are arbitrary, yet they provide a useful heuristic.

**Table 1:** IQWIG guidance [5] on interpretation of validation studies of surrogate outcomes

Strength of association	R-value
Validity proven	Lower limit of 95% CI ≥ 0.85
Unclear validity	R < 0.85 to > 0.7
Proven lack of validity	Upper limit of 95% CI ≤ 0.7

The R-value is the correlation coefficient derived through methods such as Pearson product moment, Kendall’s Tau or Spearman’s rank correlation coefficient

IQWIG defines that a surrogate has a proven lack of validity if the upper bound of the R-value’s 95% confidence interval is ≤ 0.7 [5]. Although an R-value of 0.7 might seem impressive, the corresponding R^2^ is 0.49, meaning that only 49% of the variation in survival is explained by variation in the surrogate. IQWIG considers that surrogates only have proven validity when the association demonstrates an R-value with a lower bound of the 95% confidence interval ≥ 0.85 [5]. One well-validated surrogate is disease-free survival (DFS) as a predictor of OS in adjuvant colon cancer. This surrogate was validated by examining 15 adjuvant RCTs. Plotting the hazard ratios for DFS and OS gave an R^2^_trial_ value of 0.9, suggesting that DFS was a faithful predictor for OS effects [6].

However, the reality is that many surrogates used in clinical practice do not meet this mark. In a systematic review of studies that attempt to validate surrogate outcomes, 65 specific surrogate survival pairs were identified. Of these, 52% (34) were classified as low strength (R ≤ 0.7), 25% (16) as medium strength, and only 23% (15) correlated highly (R ≥ 0.85) with OS [7]. This systematic review used an even more permissive classification than IQWIG guidance, as the point estimate of the R^2^_trial_ statistic, not the bounds of the 95% confidence interval, was used to classify the correlations as low, medium or high.

Not only are most surrogates in oncology poor predictors of survival, those used for regulatory purposes also have a weak evidence base. Of 55 regulatory approvals made by the FDA on the basis of improvements in surrogates between 2009 and 2014, 65% (36) had no trial-level validation studies. Of the 35% (18) that were studied, only 16% (3) correlated highly with survival [2].

The FDA grants drugs traditional or accelerated approval. Accelerated approval can be given based on a surrogate benefit that is ‘reasonably likely to predict’ true clinical efficacy in survival or QoL. Traditional approvals are granted when a drug demonstrates benefit in ‘established’ surrogate endpoints. However, of 25 drugs approved through the accelerated pathway, 84% (21/25) had no trial-level validation studies at all. Where validation studies were performed, correlations were demonstrated in only 14% (4/25), with R-values ≤ 0.7. Of all traditional approvals based on a surrogate, only 10% (3/30) were approved on the basis of a surrogate that had a correlation with an R-value ≥ 0.85. Of the remaining 90% (27/30) of drugs, 50% (15/30) had no trial-level validation studies, 27% (8/30) were approved on the basis of a surrogate with an association with an R-value ≤ 0.7, and 13% (4/30) with an association with an R-value between 0.7 and 0.85 [2].

While the R^2^ cut-offs used here are arbitrary, it is notable that for most approvals, no R^2^ can be calculated because no validation study has ever been done. Nevertheless, an alternative approach to validate surrogates is the use of the surrogate threshold effect, where some numerical gain in the surrogate is shown to be strongly predictive of some improvement in survival [8]. For instance, PFS may not be an ideal predictor of OS, but perhaps a PFS gain of greater than 5 months or 50% is reliable. These studies, however, are plagued by multiplicity. Since, a priori, we do not know what amount of PFS gain will be predictive, many values are explored until one, by chance alone, yields a strong correlation.

### All surrogate–survival association studies are based on a fragment of the evidence

Like all systematic reviews and meta-analyses regarding medical interventions, trial-level meta-analysis for surrogate validation should be based on an exhaustive summary of the literature relevant to the surrogate.

However, examination of trial-level meta-analyses reveals that most are based on a fragment of the evidence. Only 5/36 (14%) attempted an exhaustive literature search including published articles, abstracts, and an attempt to gain unpublished reports [7]. In those five cases, 684 trials contained data relevant to validation of the surrogate outcome, but information was available and extracted in only 51.5% of cases (352 trials). The lack of available data can bias any attempt to validate surrogate outcomes, because data that is easily located and included in surrogate validation may have different correlations than unavailable or unreported data. It is partly for this reason that we believe surrogates should only be used when confirmatory studies are ongoing, even when the correlation with outcomes remains strong. To date, no example of a surrogate validation study based on all of the relevant evidence exists. The FDA continues to attempt to validate surrogates based solely on data submitted to the agency [9], leaving open the question of whether the correlation is similar for unavailable data.

### Common justifications and benefits for using surrogates may be doubted

Surrogates are used because they are designed to be easier and quicker to measure than the hard endpoints they predict. This ostensibly enables trials to be conducted quickly, at a reduced cost, and to speed up the drug approval process. The industry has frequently criticized regulatory bodies for being too slow to grant approvals, and increased acceptance of surrogate outcomes has been proposed to remedy this [10].

However, the use of surrogates as a means to speed up drug approval is uncertain, and may lead to unintended consequences. To use validated surrogates, many trials must first be conducted on a question to validate that surrogate. Practically, this takes time, diminishing the purported benefit of using the surrogate. Second, the availability of surrogates has likely led manufacturers to alter the way in which they test newer agents, and in which populations they are tested, thus trading-off speed of approval for a larger initial market share and undermining the value of the surrogate.

Consider this scenario: traditionally, newer agents were tested in populations with more advanced disease states, e.g. relapsed and refractory cancers. These populations were increasingly likely (per unit of time) to experience the event of interest, typically death, and thus the trial duration was relatively short. Subsequent trials would then seek to confirm activity in earlier disease states where the event rate was lower, and thus trial duration was longer [11]. As surrogates become widely accepted by regulatory agencies, manufacturers have two options:

1. To conduct a trial in the same late-stage population using surrogates. In this scenario, the surrogate event will occur more quickly, meaning the trial can be conducted more quickly, and thus approval can be faster, as was claimed by many observers.
2. To conduct a trial using the surrogate but in an earlier disease state. In this scenario, the surrogate event rate may be comparable to the hard event rate in the late-stage population. This means the time to complete both study and approval is comparable. However, the earlier disease state represents a greater proportion of the market share, so the use of surrogates to speed up drug approval is traded off for grabbing a larger initial market share (Table 2).

**Table 2:** Effect of surrogate outcomes on options for designing pivotal clinical trials

	Traditional	Scenario 1: speed up drug approval	Scenario 2: increased market share	TDM-1	Pertuzumab
Population	Relapsed	Relapsed	Newly Diagnosed	2^nd^ line	1^st^ Line
Market share of population	Small	Small	Large		
Outcome	Hard	Surrogate	Surrogate	OS	PFS
Event rate	100% experience event in 1 year	100% experience event in 6 months	100% experience event in 1 year	OS benefit demonstrated after 16 months of follow-up	PFS benefit not demonstrated until 19.3 months
Time to complete study	1 year	6 months	1 year	42 months between enrollment and results	40 months between enrollment and results

*OS* overall survival, *PFS* progression-free survival

There are suggestions that this precise scenario occurred when comparing the initial approval of TDM-1 and pertuzumab, drugs tested in metastatic breast cancer that target HER2. TDM-1 – tested in the traditional manner, in the second-line setting – was approved based on an OS benefit. Between enrollment and analysis at a pre-specified cut-off time, this took 44 months to become evident [12]. In contrast, pertuzumab was initially tested in the front-line setting with a primary outcome of PFS, and took 40 months between enrollment and analysis [13]. This suggests that the time taken to conduct the studies and approve the drugs was comparable, but the initial market share for first-line therapy is certainly larger. This means that the use of surrogates may be changing the way companies seek drug approval. As companies trade off speed for market share, the role of surrogates as speeding up the process may in fact slow it down or at least maintain the current pace.

### ‘Unmet medical need’ is an overused and imprecise term

The term ‘unmet medical need’ allows the FDA to utilize accelerated approval (i.e. approval based on a surrogate) and other expedited pathways [14–17], and the FDA guidance on the term is imprecise. Recently, empirical analysis of the term in the biomedical literature suggests that it is being overused. Lu et al. found 237 cancer indications were described as ‘unmet medical need’ [18]. In 55/237 (23%) cases, ‘unmet need’ referred to indications with an annual incidence > 1000 cases, with ≥ 5 National Comprehensive Cancer Network (NCCN)-recommended regimes and a 50% greater than 5-year survival. Forty-three mentions (18%) referred to indications with an annual incidence > 10 000, 10 recommended regimes, and a 5-year survival rate of more than 50%. These results highlight that there is little professional consensus about unmet need. Thus, the use of surrogates through pathways such as accelerated approval, is likely far greater than conditions with true unmet needs.

### We have been wrong before

Recent studies have challenged the assumption that frequently used surrogates can accurately predict the effect of treatments on hard outcomes [7].

In 2008, bevacizumab was approved in combination with paclitaxel for metastatic HER2-negative breast cancer on the basis it improved PFS by 5.9 months [19]. However, subsequent confirmation studies demonstrated no benefit in OS or QoL, and found substantially increased toxicity [20]. As a result, market authorization was withdrawn in 2011.

Similar findings have been seen when pathological complete response (pathCR) is used as a surrogate outcome for OS. In a trial-level meta-analysis of 12 international trials, little association was found between frequency of pathCR and OS (R^2^ = 0.24) [21].

However, despite this weak association, pertuzumab was approved in the neoadjuvant setting on the basis of pathCR rates achieved in an RCT. The adjuvant trial of pertuzumab has now been reported, and showed a miniscule improvement in invasive disease-free survival –another surrogate – and no difference in OS [22]. At the same time, the drug is associated with increased cardiotoxicity. For this reason, experts have been critical of the use of pertuzumab in this setting [23], and pathCR may be considered a failed surrogate.

These examples emphasize that poorly validated surrogate outcomes can be misleading with regards to benefit. Premature regulatory decisions can result in patients being exposed to considerable harm for no benefit.

### We do a poor job of postmarketing follow up

Even uncertain surrogates may not be problematic if postmarketing studies of drugs approved on the basis of surrogate endpoints subsequently and reliably demonstrated benefits in meaningful outcomes. However, for most approvals this is not the case. Between 2004 and 2008, 36 oncology drugs were approved on the basis of a surrogate outcome [24]. With a median follow up of 4.4 years, 18 (50%) failed to improve OS in subsequent trials, 13 (36%) drugs continued to have unknown survival effects, and only 5 (14%) demonstrated an improvement in OS in an RCT [24].

It is often argued that treatments that fail to improve OS but do improve surrogate outcomes may still have meaningful clinical benefit, either by improving QoL, or being more cost-effective. However, in many cases there is insufficient evidence to reliably make such assertions. The 18 drugs approved that failed to demonstrate a benefit in OS were examined to see if they improved QoL. Only 1 (5%) demonstrated improved QoL, 6 made no statistical difference, 2 were associated with worse QoL, 4 with mixed results and 5 had no evidence concerning QoL [25]. This meant that 47% (17/36) of the drugs approved on the basis of surrogate outcomes, which are now routinely used in the clinic, have no clear benefit in either OS or QoL. Only one of these drugs has had its marketing authorization removed.

### There are two major reasons why surrogates may fall short

Surrogates may fall short in their ability to predict outcomes in hard endpoints for two broad categories of reasons: (1) technical factors in measuring the surrogate introduce such uncertainty that their association with the hard endpoints becomes weak; and (2) something about the relationship between the surrogate, hard endpoint and drug weakens a direct causal link between the surrogate and the hard endpoint.

In some cases, measuring surrogate endpoints can be more complex than measuring hard endpoints. Factors such as measurement error, evaluation bias, attrition bias or informative censoring may weaken the association between the surrogate and hard end point such that its predictive value is lost.

The BOLERO-2 study, which examined the efficacy of everolimus in hormone receptor-positive advanced breast cancer, demonstrated an improvement in PFS by 6.5 months [26], but failed to demonstrate any improvement in OS [27]. Informative censoring may have occurred more frequently in the treatment arm than in the control arm. Informative censoring means that the probability a person was censored is related to the probability of experiencing the outcome. Specifically, if censoring occurs more often in patients who experience early toxicity, and if the presence of toxicity is more likely to be experienced by patients with limited physiologic reserve or more aggressive cancer biology, then censoring these patients may lead to a false overestimation of the Kaplan–Meier curve, as the sickest patients are removed. If informative censoring is differential (occurs more often in the treatment than in the control group), then the efficacy of the intervention may be falsely inferred. Informative censoring fundamentally violates the Kaplan–Meier assumption. Both informative and differential censoring may have occurred in BOLERO-2, as everolimus has marked toxicity and high rates of dose reduction. Some patients censored initially on this arm were almost surely censored for toxicity. In an independent analysis, we have shown that the PFS benefit in BOLERO-2 can vanish if one alters the assumptions around censoring [28].

Even if a surrogate is perfectly measured it may not be able to predict hard endpoints if there is something in the relationship between the surrogate, drug and hard endpoint that undermines the drug’s benefit; for example, the surrogate may not have a causal role in the hard endpoint. This may arise if, for instance, pathCR does not relate to OS because of micrometastatic disease outside the resection areas responsible for OS, which are not measured by the surrogate. Alternatively, this might arise if an intervention has offsite target effects that are independent of the disease process. This can affect the hard endpoint, or whether post-protocol disease growth is accelerated, as well as other possibilities.

## Where do we go from here?

Many of the commonly used surrogates have known the problems described above. However, improved standardization may permit novel surrogates to be identified that have the potential for greater predictive power [29].

Retrospective analysis of castration-resistant prostate cancer trials allowed a growth constant, g, to be defined based on prostate-specific antigen-level dynamics [30]. In clinical trials, g was associated with patient survival between arms and could predict a benefit in survival (at 80% power) with only 50 people per arm.

Robust surrogate outcomes have the potential to reduce the clinical, economic and time burden associated with RCTs and drug development. However, this benefit is only achieved if the surrogate is thoroughly validated in establishing true surrogacy for meaningful outcomes, and if the industry does not use the surrogate to justify a longer study to seek a broader market share. Moreover, validation of both existing and novel surrogates may take years, undermining the speed of surrogates. Failure to validate surrogates may lead to harmful drugs entering and persisting on the market.

## Conclusions

The factors outlined here lead us to conclude that surrogates should lead to practice change or drug approval only when robust validation studies demonstrate that a change in a specific surrogate has a reliable ability to predict changes in meaningful outcomes. Here we favor strong correlations in line with the IQWIG thresholds. Alternatively, surrogates may also be used when not validated in conditions that are rare, dire, and with few treatment options. In both cases, surrogates should be used after studies measuring hard endpoints have been fully recruited, and are ongoing. We believe this standard balances the benefits of surrogates with the importance of strict regulatory standards. Future work should further explore the unintended consequences of the use of surrogate endpoints in oncology.
